# Immunogenicity of yellow fever vaccine co-administered with 13-valent pneumococcal conjugate vaccine in rural Gambia: A cluster-randomised trial

**DOI:** 10.1016/j.vaccine.2025.126712

**Published:** 2025-02-15

**Authors:** Isaac Osei, Jonas Schmidt-Chanasit, Paul V. Licciardi, Ousman Secka, Umberto D'Alessandro, Rasheed Salaudeen, Golam Sarwar, Ed Clarke, Nuredin I. Mohammed, Cattram Nguyen, Brian Greenwood, Stephanie Jansen, Grant A. Mackenzie

**Affiliations:** a Medical Research Council Unit The Gambia at London School of Hygiene & Tropical Medicine, Banjul, the Gambia; b Department of Disease Control, Faculty of Infectious and Tropical Diseases, London School of Hygiene & Tropical Medicine, London, UK; c Bernhard Nocht Institute for Tropical Medicine, Department of Arbovirology and Entomology, Hamburg, Germany; d Murdoch Children's Research Institute, Melbourne, Australia; e Department of Paediatrics, University of Melbourne, Australia; f University of Hamburg, Faculty of Mathematics, Informatics and Natural Sciences, Hamburg, Germany

**Keywords:** Pneumococcal conjugate vaccine, Yellow fever vaccine, Co-administration, Neutralizing antibody titre, The Gambia

## Abstract

**Introduction:**

Because booster doses of pneumococcal conjugate vaccine (PCV) may be given at a similar time to yellow fever vaccine (YF), it is important to assess the immune response to YF when co-administered with PCV. This has been investigated during a reduced-dose PCV trial in The Gambia.

**Methods:**

In this phase 4, parallel-group, cluster-randomized trial, healthy infants aged 0–10 weeks were randomly allocated to receive either a two-dose schedule of PCV13 with a booster dose co-administered with YF vaccine at age 9 months (1 + 1 co-administration) or YF vaccine administered separately at age 10 months (1 + 1 separate) or the standard three early doses of PCV13 with YF vaccine at age 9 months (3 + 0 separate). Blood samples were collected 28–35 days post-vaccination and YF neutralizing antibody (NA) titres were measured. Proportions with seroprotective YF NA titres ≥ 1:8 were calculated with 95 % confidence intervals (CI). Non-inferiority was demonstrated if the lower limit of the CI for the difference in proportions between the co-administration and separate groups was greater than − 10 %.

**Results:**

Forty-eight, 66, and 98 participants enrolled in 3 + 0 separate, 1 + 1 co-administration, and 1 + 1 separate groups respectively had NA results. Per protocol analysis of the 3 + 0 separate, 1 + 1 co-administration, 1 + 1 separate, and the combined 1 + 1 separate and 3 + 0 separate groups found that 81 %, 85 %, 92 %, and 88 % of participants respectively had YF NA titres ≥1:8. Results were similar with analysis by intention-to-treat. The difference in proportions comparing 1 + 1 co-administration and 1 + 1 separate groups was −7 % (95 % CI, −18 % to 3 %). The difference between 1 + 1 co-administration and 3 + 0 separate groups was 4 % (95 % CI, −10 % to 15 %). There was no statistical difference in the YF seroresponse when the YF vaccine was co-administered with PCV or administered separately.

**Conclusions:**

No evidence was found of the non-inferiority of the seroresponse to YF vaccine when co-administered with PCV13. The levels of YF NA attaining seroprotection (NT ≥1:8) were high in all groups. PCV13 co-administered with YF vaccine at 9 months does not affect seroresponse to YF vaccine. http://www.isrctn.org/ - ISRCTN72821613.

## Highlights

• Overall, 86 % of participants had YF NA titres ≥1:8 at 1 month (mo) post-YF vaccination (YFV)
• 81 %, 92 %, & 85 % of infants who received YFV separately at 9 & 10 mo, and YFV/PCV at 9 mo respectively had YF NA titres ≥1:8
• PCV13 co-administered with YF vaccine at 9 mo does not affect seroresponse to YF vaccine.
• The difference in proportions comparing YFV/PCV co-administered at 9 mo and YFV given separately at 10 mo was −7 %
• There was no statistical difference in the YF seroresponse when the YFV was co-administered with PCV or administered separately

## 1 Introduction

Yellow fever (YF) is caused by an orthoflavivirus endemic in Africa, Central America, and South America. It is estimated that in 2018, 109,000 people experienced severe disease with YF infection, with 51,000 deaths in Africa and South America. [1] Between January 2023 and February 2024, 13 countries in Sub-Saharan Africa documented probable or confirmed cases of YF, with an 11 % case fatality rate. [2] A single dose of the live-attenuated YF vaccine provides effective immunity to over 99 % of vaccinated persons within 30 days and confers lifelong protection. [[3], [4], [5]] A booster dose of YF vaccine is recommended for travellers who plan to spend a prolonged period or travel to highly endemic areas and who received their last dose of the YF vaccine at least 10 years previously. [6]

In 2024, 194 World Health Organization (WHO) member countries and 25 countries in the WHO African Region introduced the YF vaccine into their routine Essential Programme on Immunization (EPI) programs. [7] YF vaccine is given as a single dose, generally at 9 months of age, and often co-administered with measles/rubella vaccine and oral polio vaccine. Although YF vaccine is safe when co-administered with measles/rubella vaccine, there is inconsistent evidence regarding its effect on YF immunogenicity. [8] While some studies have shown no decrease in YF vaccine seroresponse when co-administered with measles/rubella vaccines, [9,10], other studies have reported a decreased seroresponse against YF vaccines. [[11], [12], [13]]

The introduction of pneumococcal conjugate vaccines (PCVs) into routine immunization programmes has led to a substantial decrease in the global incidence of pneumococcal diseases attributable to vaccine serotypes (VT). [[14], [15], [16], [17]] However, the relatively high cost of PCV impedes its introduction in many middle-income countries and its cost is an ongoing factor in the sustainability of PCV programmes in most developing countries. [18] Currently, WHO recommends a three-dose schedule for PCV, given either as three doses in early infancy (i.e. a 3 + 0 schedule) or two early doses with a later booster dose at 9–18 months of age (2 + 1 schedule). [19] An estimated 57 countries, mainly high-income, use the 2 + 1 PCV schedule while most low-middle-income countries use the 3 + 0 schedule. [20] Decreasing the number of PCV doses without compromising effectiveness has been suggested as an approach to facilitate the sustainability of PCV programmes. Including booster doses, while reducing the total number of doses of PCV, may be possible in countries with mature vaccination programmes in which herd effects have assumed a greater influence than direct effects. [21] There is, therefore, a global initiative to generate evidence about reduced-dose PCV schedules. An important question for using PCV booster doses in YF endemic countries is the immunogenicity of the YF vaccine when co-administered with PCV, which in Africa is usually scheduled for administration at 9 months of age. Evidence concerning the immunological response when PCV and YF are co-administered will assist policymakers in considering recommendations for the scheduling of PCV booster doses in a 2 + 1 schedule or a reduced-dose schedule of two doses, with one early dose and one booster dose (i.e. a 1 + 1 schedule). We report the immunogenicity of YF vaccine when co-administered with PCV13 in a reduced-dose PCV trial in The Gambia.

## 2 Methods

### 2.1 Study design and participants

This study of pneumococcal vaccine schedules, acquisition and immunogenicity (PVS-AcqImm) [22] was nested within a large Pneumococcal Vaccine Schedules (PVS), cluster-randomized trial of PCV scheduling which is ongoing in the Central (CRR) and Upper River Regions (URR) of The Gambia. Details of the objectives of this trial and the methods employed have been presented previously. [23] PVS-AcqImm is a parallel-group, cluster-randomized trial of the individual-level effect of two different schedules of PCV13. [22] Infants resident in the 28 PVS-AcqImm geographic clusters of villages and aged 0–10 weeks received PCV13 (Prevnar-13, manufactured by Pfizer Ltd., New York City, USA) in either the 3 + 0 standard schedule with doses given at ages of 6, 10 and 14 weeks, with YF vaccine administered at 9 months of age (3 + 0 PCV/YF separate 9-month), or an alternative 1 + 1 schedule of PCV13 with doses scheduled at ages 6 weeks and 9 months. Participants in the 1 + 1 schedule clusters who were assigned to the measurement of immunogenicity endpoints were further randomly assigned to receive the YF [17D-204 YF vaccine, manufactured by Institut Pasteur Dakar, Senegal] vaccine [24] co-administered with PCV13 at 9 months of age (1 + 1 PCV/YF co-administration 9-month group) or administered separately at 10 months of age (1 + 1 PCV/YF separate 10-month group). As per the Gambian EPI schedule, all participants received the measles/rubella vaccines at 9 months of age. The PCV13 and YF vaccines were delivered in collaboration with, and through the structures of the Gambian EPI within the operational framework of the public health system. Blood samples were collected 28–35 days post-YF vaccine administration. Aliquots of serum were stored at The Medical Research Council Unit The Gambia (MRCG) Basse Field Station at −70 °C before shipment to the Bernard Nocht Institute for Tropical Medicine (BNITM), Hamburg, for YF serology. The details of the inclusion and exclusion criteria are described in the study protocol and statistical analysis plan which have been published previously. [22,25]

### 2.2 Outcomes

The primary outcome was the proportion of participants with a YF NA titres ≥ 1:8 1 month after administration of YF vaccine, comparing those in the 1 + 1 PCV/YF co-administration 9-month group to those in the 1 + 1 PCV/YF separate 10-month group using a per-protocol (PP) cohort. Secondary outcomes included differences in proportions and CIs comparing the PCV/YF 9-month co-administration group to the 3 + 0 PCV/YF separate 9-month group and to the combined 1 + 1 YF/PCV separate 10-month and 3 + 0 groups, in both of which YF vaccine was administered separately without PCV. [22,25]

### 2.3 Statistical power and sample size

Power was calculated using a formula for non-inferiority tests for two proportions using Nquery + nTerim software and inflated to account for non-independence of results within clusters (ICC = 0.01, average cluster size = 8). The sample size calculation assumed a one-sided alpha = 0.025, beta = 0.9, a baseline proportion of 95 % with YF NA titres ≥1:8 [10] and a non-inferiority margin of 10 % absolute difference in proportions. With 112 participants in each group and allowing for a 5 % loss to follow-up, the study would have 90 % power to test the null hypothesis that co-administration is not inferior to separate administration.

### 2.4 Randomization and masking

At enrolment, pre-prepared computer-generated random assignment lists organized by cluster were used to assign participants to various blood collection schedules. For the 1 + 1 clusters, the pre-prepared random assignment lists also specified random assignment of individuals to the PCV/YF 9-month co-administration or 1 + 1 YF/PCV separate 10-month administration groups. Thus, the random allocation of participants to the group and blood collection schedules were electronically generated. An independent statistician prepared the cluster randomization lists. Participant's enrolment was performed by trial staff. The trial data manager prepared the list for the random allocation of participants to different blood collection schedules and to various arms of the trial. Vaccinators and parents were aware of the schedules used. Specimens were labelled with a unique identification number that did not reveal the study group, ensuring that laboratory staff were blinded. Blinding the laboratory staff prevented bias considering the study's laboratory-based objectives. Data were analysed in a pseudo-blinded fashion with the two groups identified by an indicator label rather than the identity of each group. [22,25]

### 2.5 Reactogenicity

An established electronic vaccine record system [26] was used to record unsolicited events of reactogenicity following a dose of YF vaccine reported by caregivers up to 1-month post-administration.

### 2.6 YF serology

A porcine kidney epithelial cell monolayer was cultured in 96-well plates (Sarstedt, Germany). The cells were maintained under standard conditions at 37 °C with 5 % CO₂ in a humidified incubator. Heat-inactivated sera (56 °C for 30 min) were prepared in serial two-fold dilutions ranging from 1:8 to 1:256. All dilutions were tested in duplicate (100 μl/well). [27]. For each serum dilution, 100 TCID₅₀ (median tissue culture infectious dose) units of the YF virus strain YF-17D (passage 2) were added, the resulting virus-serum solution was incubated for 1 h at 37 °C and afterwards added to the pre-cultured porcine cell monolayers. The plates were incubated under standard cell culture conditions for 7 days. Afterwards, the cells were fixed and stained for 30 min using a solution containing 4 % formaldehyde and 0.25 % (*w*/*v*) crystal violet (Roth, Germany).

Evaluation of neutralization was based on the presence or absence of a viral-induced cytopathic effect (CPE). Wells showing staining indicated protection from CPE, and wells without staining indicated the presence of CPE. The neutralization titre (NT) was determined based on the highest dilution that showed 50 % protection while presenting 100 % infection in consecutive dilutions. If a serum dilution showed 100 % protection followed by 100 % infection, the geometric mean of both serum dilutions was calculated as the NT. A discriminatory cut-off for seroprotection was set at a titre of ≥1:8 indicating a positive result and titres <1:8 were considered negative. The seroprotective threshold of YF NA titres ≥1:8 was chosen based on previous studies. [10,28,29]

### 2.7 Statistical considerations

Baseline characteristics were reported in four groups: (a) 3 + 0 PCV/YF separate 9-month, (b) 1 + 1 PCV/YF co-administration 9-month, (c) 1 + 1 PCV/YF separate 10-month, and (d) combined 1 + 1 YF/PCV separate 10-month and 3 + 0 groups, in both of which YF vaccine was separately administered. Reporting of baseline characteristics in these four groups allowed assessment of the outcomes of the two stages of randomization, i.e. 1 + 1 vs 3 + 0 and also 1 + 1 PCV/YF separate 10-month vs 1 + 1 PCV/YF co-administration 9-month groups. The primary endpoint of seroprotection 1 month after YF vaccination in 1 + 1 PCV/YF separate 10-month, and 1 + 1 PCV/YF co-administration 9-month groups was compared using a per-protocol (PP) cohort. Participants were excluded from the PP analysis if i) assigned to the 1 + 1 PCV/YF separate 10-month group but inadvertently received the 1 + 1 PCV/YF co-administration 9-month group schedule ii) assigned to 3 + 0 PCV/YF separate 9-month or 1 + 1 PCV/YF co-administration 9-month group and received YF vaccine at age < 273 days (9 months) or > 350 days (11.5 months) iii) assigned to 1 + 1 PCV/YF separate 10-month group and received YF vaccine at age < 304 days (10 months) or > 380 days (12.5 months) iv) the YF vaccination date and post-YF specimen collection date was <28 days or > 35 days apart. All participants who were excluded from the PP analysis were included in intention-to-treat (ITT) analyses, in groups as originally randomized. Proportions with YF neutralizing antibody titres ≥ 1:8 were calculated with exact binomial 95 % confidence intervals (CI). The difference in proportions and a 95 %CI was computed using a binomial model with an identity link and generalized estimating equations (GEE) with an exchangeable correlation structure, including terms for cluster identity and the cluster stratifying covariate of ‘high-low’ incidence of clinical pneumonia. For the primary analysis, the seroresponse in the co-administration group was deemed non-inferior if the lower limit of the two-sided 95 %CI for the difference in proportions (PCV/YF 9-month co-administration minus PCV/YF 10-month separate administration) was greater than − 10 %. [10,28] Secondary analyses included calculation of differences in proportions and CIs comparing the PCV/YF 9-month co-administration group to the 3 + 0 PCV/YF separate 9-month group and to the combined 1 + 1 YF/PCV separate 10-month and 3 + 0 groups, both in which YF vaccine was administered separately. [25] A sensitivity analysis adjusting for age at the time when YF vaccination was given to determine whether the difference in the age of administration of YF vaccine in the groups influenced YF immune response. Additionally, we explored whether the excluded children differed in background characteristics from those who were included in the analyses. Analyses were performed separately in PP and ITT cohorts, using Stata version 18.0.

### 2.8 Ethics

The study was approved by the Gambia Government/MRC Joint Ethics Committee (ref: 17683) and by the LSHTM Ethics Committee (ref: 17683). Written, informed consent to participate was obtained from all enrolled participants.

## 3 Results

### 3.1 Participants, enrolment, and specimens

**Fig. 1:**
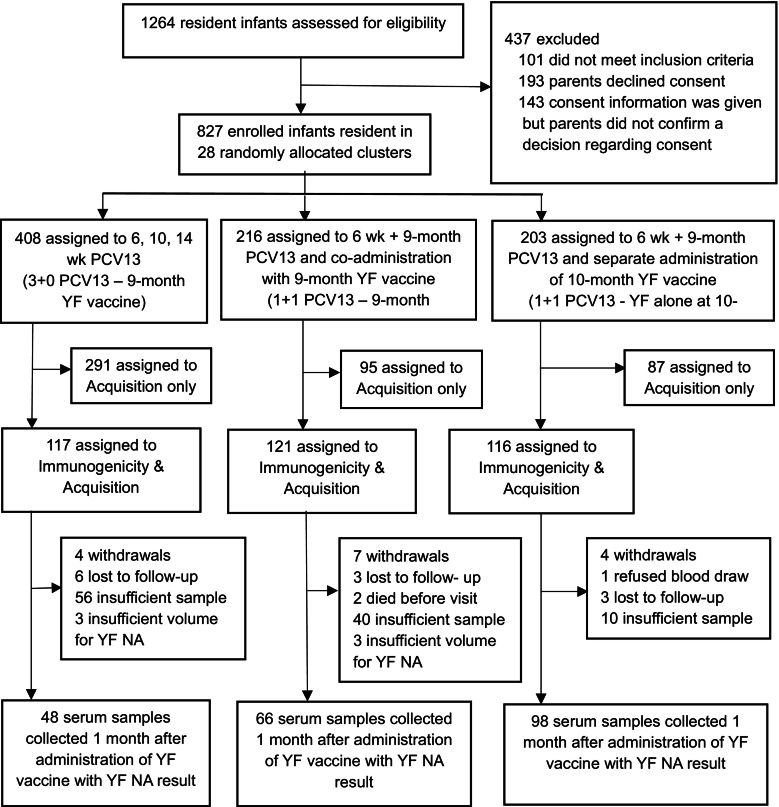
Trial Profile. Infants aged 0-10 weeks residents in 28 clusters of villages were randomly allocated to receive PCV13 delivered in either a 3+0 or 1+1 schedule. Participants in the 1+1 schedule clusters who were assigned to the measurement of immunogenicity endpoints were randomly allocated to receive either the YF vaccine co-administered at 9 months of age with the PCV booster dose (1+1 PCV/YF co-administration 9-month group) or administered separately at 10 months of age (1+1 PCV/YF separate 10-month group). Blood samples were collected 28-35 days after administration of YF vaccine.

Between 14 September 2020 and 28 October 2021, 1264 infants were screened and 1163 were eligible for enrolment. Of these, 827 were enrolled. Of those enrolled, 408 were residents in clusters assigned to the 3 + 0 schedule of PCV13 and 419 were residents in clusters assigned to the 1 + 1 schedule. Two hundred and thirty-seven of those residents in 1 + 1 clusters were randomly assigned to the measurement of immunogenicity endpoints; 121 were randomly assigned to receive YF vaccine co-administered at 9 months of age with the PCV booster dose (PCV/YF 9-month co-administration group) and 116 assigned to receive YF vaccine administered separately at 10 months of age (1 + 1 YF/PCV separate 10-month group). One hundred and seventeen participants in the 3 + 0 group were randomly assigned to immunogenicity measurements. Only 48, 66, and 98 participants in the 3 + 0 PCV/YF separate 9-month, 1 + 1 PCV/YF 9-month co-administration, and 1 + 1 YF/PCV separate 10-month groups respectively (146 participants in the combined 1 + 1 YF/PCV separate 10-month and 3 + 0 groups) had valid results from blood specimens collected 28–35 days post-YF vaccine administration and were eligible for analysis. The drop in the number of participants that remained for analyses was due mainly to insufficient blood sample volumes (Fig. 1). The higher number of insufficient samples for YF NA testing noted in the 3 + 0 PCV/YF separate 9-month and 1 + 1 PCV/YF 9-month co-administration groups compared to the 1 + 1 YF/PCV separate 10-month group was due to higher volumes of samples required for additional testing of pneumococcal immunogenicity OPA and IgG measurements at the 1-month post-YF vaccination sample collection visits in the 3 + 0 PCV/YF separate 9-month and 1 + 1 PCV/YF 9-month co-administration groups. In the 1 + 1 YF/PCV separate 10-month group, the 1-month post-YF vaccination samples were collected for YF NA testing only (Supplementary table 1).

Forty-six children were excluded from the PP cohort for the following reasons - assigned to the 1 + 1 PCV/YF separate 10-month group but inadvertently received the 1 + 1 PCV/YF co-administration 9-month group schedule(*n* = 17); assigned to the 3 + 0 PCV/YF separate 9-month (*n* = 4) or 1 + 1 PCV/YF co-administration 9-month group (*n* = 3) and received YF vaccine at age < 273 days or > 350 days; assigned to the 1 + 1 PCV/YF separate 10-month group and received YF vaccine at age < 304 days (n = 4) or > 380 days (*n* = 8); the YF vaccination date and post-YF specimen collection date was <28 days or > 35 days apart (3 + 0 PCV/YF separate 9-month group (n = 1,) 1 + 1 PCV/YF co-administration 9-month group (n = 3) and 1 + 1 PCV/YF separate 10-month group (*n* = 6).

There were similar distributions of age at enrolment, PCV and YF vaccine administration, post-YF vaccination blood collection, sex, and other indicators in the four groups in PP and ITT cohorts (Table 1) as well as in the participants who were assigned to immunogenicity measurements (Supplementary table 2). The children who were excluded from the endpoint measurements had balanced baseline characteristics compared to those who were included in the ITT analysis across all groups (Supplementary table 3).

Infants aged 0–10 weeks residents in 28 clusters of villages were randomly allocated to receive PCV13 delivered in either a 3 + 0 or 1 + 1 schedule. Participants in the 1 + 1 schedule clusters who were assigned to the measurement of immunogenicity endpoints were randomly allocated to receive either the YF vaccine co-administered at 9 months of age with the PCV booster dose (1 + 1 PCV/YF co-administration 9-month group) or administered separately at 10 months of age (1 + 1 PCV/YF separate 10-month group). Blood samples were collected 28–35 days after administration of YF vaccine.

### 3.2 Proportions of participants with YF neutralization titres ≥ 1:8

Overall, the percentage of participants with YF NA titres ≥1:8 at 1 month post-YF vaccination was 86 % (95 %CI; 84.0 % – 87.0 %). In the PP cohort, the percentage of participants with YF NA titres ≥1:8 at 1 month post-YF vaccination was 92 % (82 % - 97 %) in the 1 + 1 PCV/YF separate 10-month group, 81 % (66 % - 92 %) in the 3 + 0 PCV/YF separate 9- month group, and 85 % (73 % - 93 %) in the 1 + 1 PCV/YF co-administration 9-month group (Table 2). The proportion of participants with YF NA titres ≥ 1:8 in the two combined groups who received separate administration of YF vaccine without PCV (1 + 1 PCV/YF separate and 3 + 0 PCV/YF separate groups) was 88 % (80 % - 93 %). In the ITT cohort, the proportions of participants in the 1 + 1 PCV/YF co-administration 9-month group with YF NA titre ≥ 1:8 was similar to that observed in the PP cohort, the proportions decreased by 2 % respectively in the other three groups (Table 2). There was no statistical difference in the YF seroresponse when the YF vaccine was co-administered with PCV or administered separately.

**Table 2:** Yellow Fever neutralizing antibody titres ≥ 1:8 1 month post-vaccination.

Study group	n/N	Percentage (95% CI) participants Yellow Fever neutralizing antibody titre ≥1:8	Difference in percentages (95% CI) compared to YF/PCV co-administration
*Per Protocol (PP)*			
1+1 PCV/YF co-administration 9-month	51/60	85% (73% to 93%)	1 (reference)
3+0 PCV/YF separate 9-month	35/43	81% (66% to 92%)	4% (-10% to 15%)
1+1 PCV/YF separate 10-month	58/63	92% (82% to 97%)	-7% (-18% to 3%)
1+1 PCV/YF separate and 3+0 PCV	93/106	88% (80% to 93%)	-3% (-15% to 6%)
*Intention to treat (ITT)*			
1+1 PCV/YF co-administration 9-month	56/66	85% (74% to 92%)	1 (reference)
3+0 PCV/YF separate 9-month	38/48	79% (65% to 90%)	6% (-8% to 17%)
1+1 PCV/YF separate 10-month	88/98	90% (82% to 95%)	-5% (-13% to 3%)
1+1 PCV/YF separate and 3+0 PCV	126/146	86% (80% to 91%)	-1% (-12% to 6%)

ITT – Intention to treat.

PP – Per protocol.

3 + 0 PCV/YF separate 9-month– three early doses of PCV13 scheduled at 6,10, and 14 weeks and Yellow Fever/Measles/Rubella vaccines at 9 months of age.

1 + 1 PCV/YF co-administration 9-month– PCV13 was given at 6 weeks and Yellow Fever vaccine was given together with PCV13 and Measles/Rubella vaccines at 9 months of age.

1 + 1 PCV/YF separate 10-month– PCV13 was given at 6 weeks and 9 months and Yellow Fever vaccine was given separately at 10 months of age.

1 + 1 PCV/YF separate and 3 + 0 PCV– Yellow fever vaccines were given separately at 10 months and 9 months of age respectively without PCV13.

**Table 1:** Baseline characteristics of participants included in per protocol and intention to treat analysis, by group.

Characteristic	Group							
	3 + 0 PCV/YF separate 9-month		1 + 1 PCV/YF co-administration 9-month		1 + 1 PCV/YF separate 10-month		1 + 1 PCV/YF separate and 3 + 0 PCV	
	PP	ITT	PP	ITT	PP	ITT	PP	ITT
No. enrolled	43	48	60	66	63	98	106	146
Age at enrolment (days), n	43	48	60	66	63	98	106	146
median (IQR), n	19 (11−33)	21 (11–33)	22 (15–35)	22 (15–34)	23 (17–43)	27 (17–43)	22 (14–37)	24 (16–39)
Sex, n	43	48	60	66	63	98	106	146
female, n (%)	22 (51 %)	23 (48 %)	29 (48 %)	30 (45 %)	33 (52 %)	53 (54 %)	55 (52 %)	76 (52 %)
Gestational age at birth, n	43	48	60	66	63	98	106	146
median (IQR)	38 (37–38)	38 (37–38)	38 (37–38)	38 (37–38)	38 (37–38)	38 (37–38)	38 (37–38)	38 (37–38)
^#^Birth weight, n	33	37	48	52	53	86	86	123
median (IQR), n	3.0 (2.8–3.2)	3.0 (2.8–3.3)	3.1 (3.0–3.5)	3.1 (3.0–3.5)	3.0 (2.7–3.4)	3.0 (2.7–3.3)	3.0 (2.8–3.3)	3.0 (2.8–3.3)
Breastfed at enrolment, n	43	48	60	66	63	98	106	146
yes, n (%)	43 (100 %)	48 (100 %)	60 (100 %)	66 (100 %)	63 (100 %)	98 (100 %)	106 (100 %)	146 (100 %)
Age at first PCV dose (days), n	43	48	60	66	63	98	106	146
median (IQR), n	61 (50–68)	60 (50–67)	55 (50–64)	55 (50–63)	55 (48–64)	56 (48–65)	56 (48–65)	56 (49–65)
Age at second PCV dose (days), n	43	48	60	66	63	98	106	146
median (IQR), n	91 (82–101)	90 (83–101)	293 (282–305)	295 (283–311)	293 (285–301)	292 (283–301)	281 (99–296)	283 (101–297)
Age at third PCV dose (days), n	43	48	NA	NA	NA	NA	NA	NA
median (IQR), n	127 (118–144)	127 (118–144)	NA	NA	NA	NA	NA	NA
Age at YF vaccine dose (days), n	43	48	60	66	63	98	106	146
median (IQR), n	292 (283–307)	292 (284–310)	294 (283–305)	296 (284–311)	329 (320–351)	327 (310–356)	319 (295–334)	319 (290–348)
Antibiotics since birth, n	42	48	60	66	63	98	105	146
yes, n (%)	4 (9 %)	4 (8 %)	1 (2 %)	1 (1 %)	3 (5 %)	6 (6 %)	7 (7 %)	10 (7 %)
Smoker in house, n	42	47	60	66	63	98	105	145
yes, n (%)	2 (5 %)	3 (6 %)	10 (17 %)	11 (17 %)	9 (14 %)	14 (14 %)	11 (10 %)	17 (12 %)
Household cooking fuel, n	39	44	57	63	54	89	93	133
wood, n (%)	39 (100 %)	42 (95 %)	54 (95 %)	60 (95 %)	53 (98 %)	87 (98 %)	92 (99 %)	129 (97 %)
charcoal, n (%)	0 (0 %)	2 (5 %)	3 (5 %)	3 (5 %)	1 (2 %)	2 (2 %)	1 (1 %)	4 (3 %)
Infant inside cooking area sometimes, n	39	44	57	63	54	89	93	133
yes, n (%)	19 (49 %)	21 (48 %)	30 (53 %)	35 (55 %)	25 (46 %)	47 (53 %)	44 (47 %)	68 (51 %)
Age post YF vaccine blood collection (days), n	43	48	60	66	63	98	106	146
median (IQR), n	317 (313–324)	318 (313–335)	327 (310−331)	328 (315–364)	359 (344–360)	360 (349–380)	347 (334–359)	359 (337–373)

# These births occurred at home, so newborns were not weighed at birth.

ITT – Intention to treat; PP – Per-protocol; NA; Not Applicable.

3 + 0 PCV/YF separate 9-month– Three early doses of PCV13 scheduled at 6,10, and 14 weeks and Yellow Fever/Measles/Rubella vaccines at 9 months of age.

1 + 1 PCV/YF co-administration 9-month– PCV13 was given at 6 weeks and Yellow Fever vaccine was given together with PCV13 and Measles/Rubella vaccines at 9 months of age.

1 + 1 PCV/YF separate 10-month– PCV13 was given at 6 weeks and 9 months and Yellow Fever vaccine was given separately at 10 months of age.

1 + 1 PCV/YF separate and 3 + 0 PCV– Yellow fever vaccines were given separately at 10 months and 9 months of age respectively without PCV13.

### 3.3 Non-inferiority analyses of YF neutralizing titres

**Fig. 2:**
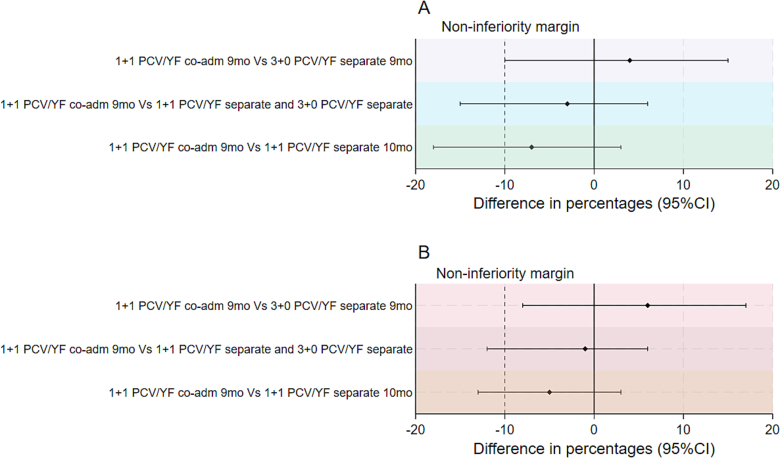
Non-inferiority analyses of YF virus neutralizing antibody titres in (a) per-protocol and (b) intention to treat analyses.

The lower limit of the CI for the difference in proportions comparing the 1 + 1 PCV/YF co-administration 9-month group to the 1 + 1 PCV/YF separate 10-month group in both the PP and ITT analyses was less than − 10 %. i.e. (−18 % and − 13 % respectively). Therefore, non-inferiority of the immune response of the YF vaccine when co-administered with PCV13 was not demonstrated (Fig. 2). The immune response to the YF vaccine in the 1 + 1 PCV/YF co-administration 9-month group, although with wide confidence limits, was higher compared to the immune response in the 3 + 0 PCV/YF separate 9-month group in which children received the YF vaccine separately together with measles/rubella vaccine at 9 months of age. The lower limit of the CI for the difference in proportions comparing the 1 + 1 PCV/YF co-administration 9-month group to all the children who received the YF vaccine separately i.e. (1 + 1 PCV/YF separate and 3 + 0 PCV/YF separate groups) in both the PP and ITT cohort was less than − 10 % i.e. (−15 % and − 12 % respectively). The sensitivity analysis adjusted for age at YF vaccination showed similar results in the PP and ITT cohorts (Supplementary table 3).

The solid vertical line indicates 0 % difference and dashed vertical lines show the inferiority margins at −10 % difference in proportions, and the horizontal lines show the point estimates and 95 %CIs for 1 + 1 PCV/YF co-administration given at 9-month compared with i) 3 + 0 PCV/YF separate given at 9 months ii) 1 + 1 YF/PCV separate given at 10 months and 3 + 0 PCV/YF separate given at 9 months iii) 1 + 1 YF/PCV separate given at 10 months.

## 4 Discussion

This study evaluated the immunogenicity of YF vaccine when co-administered with PCV13 at 9 months of age compared to the standard separate administration. The primary results showed that the lower limit of the CI for the difference in proportions comparing the 1 + 1 PCV/YF co-administration 9-month to the 1 + 1 PCV/YF separate 10-month group in the PP analyses was less than the non-inferiority margin of −10 % (difference in proportions −7 % [−18 %, 3 %]) with wide confidence intervals. In the secondary analysis for both the PP and ITT cohorts, the immune response to the YF vaccine in the 1 + 1 PCV/YF co-administration 9-month group, although with wide confidence intervals, was higher compared to the 3 + 0 PCV/YF separate 9-month group in which children received the YF vaccine with measles/rubella vaccine at 9 months (difference in proportions 4 % [−10, 15 %] and 6 % [−8 %, 17 %] respectively). The difference in proportions comparing the 1 + 1 PCV/YF co-administration 9-month group and the two groups combined who received separate administration of the YF vaccine without PCV (1 + 1 PCV/YF separate 10-month and 3 + 0 PCV/YF separate groups) was −3 % with wide confidence intervals (−15 %, 6 %). Given the inconsistent directions of effect in these analyses and the wide confidence intervals and reduced power due to missing data, the study was unable to generate conclusive evidence to test the initial hypothesis. However, the evidence obtained suggests that the levels of YF NA attaining seroprotection (NT ≥1:8) were high and robust in all groups. PCV13 co-administered with YF vaccine at 9 months does not affect the seroresponse to YF vaccine adversely.

One month post-vaccination, 85 % of children in the 1 + 1 PCV/YF co-administration 9-month group and 92 % in the 1 + 1 PCV/YF separate 10-month group had attained YF seroprotection (NA titre ≥1:8). This finding is consistent with previous studies which showed seroconversion to YF-17D vaccines in healthy infants ranged from 70 % to 89 %. The immune responses of YF vaccine are generally lower in young children than in older children and adults(mostly >95 %) [11,28,[30], [31], [32], [33]]. Although the clinical implications of this are still not well defined. Consequently, some YF endemic countries such as Brazil have introduced a second dose administered at 4 years to address this concern. [34,35]

Our findings corroborate previous studies that have sought to assess the immunogenicity of PCV when co-administered with YF vaccine. Both studies were conducted in The Gambia. The more recent study evaluated the immunogenicity of a novel 10-valent PCV in healthy infants. PCV-naive infants aged 6–8 weeks received either the 10-valent PCV (SIIL-PCV; Serum Institute of India; Pune, India) or pneumococcal polysaccharide-protein D-conjugate vaccine (PHiD-CV; Synflorix; GlaxoSmithKline; Brentford, UK) co-administered with EPI vaccines. The proportions of YF NA titres ≥1:8 in which YF vaccines were administered at 9 months were 99.1 % and 96.6 % in the SIIPL-PCV + YF vaccine group and the PHiD-CV + YF vaccine group respectively [36]. In contrast to our study, which compared the immune response of the 1 + 1 PCV/YF co-administration 9-month group to the 1 + 1 PCV/YF separate 10-month group, the study did not have a YF vaccine administered separately comparator, so it was not possible to test whether the response to YF vaccine was influenced by co-administration. In our study, in the 3 + 0 PCV/YF separate 9-month group in which YF vaccine was given separately at 9 months of age (with measles/rubella vaccine), the proportion of participants with YF NA titre ≥1:8 at 10 months of age was surprisingly low, at 79 % in the ITT cohort and 81 % in the PP cohort. In the previously mentioned Gambian study in which the investigators controlled the administration of all vaccines, the seroresponse to the YF vaccine was >95 %.

A similar study in the Gambia which evaluated the immunogenicity of PCV formulations containing PCV10 (PHiD-CV) and two conserved pneumococcal proteins in two schedules, 3 + 0 and 2 + 1, found that the co-administration with YF vaccine did not decrease the immune response of the YF vaccine. These results showed that infants assigned to the 3 + 0 and the 2 + 1 groups had antibody levels greater than the seroprotective level for YF neutralizing antibody titre >1:10 (96.9–100 % and 95.8–97.9 % respectively) when measured 3 months post-administration of PCV + YF vaccine. [37] In contrast to both studies mentioned above in which investigators controlled the storage and the administration of all vaccines, in our study the delivery of the vaccines was through the existing government EPI structures, reflecting real-world immunization scenarios. Therefore, the lower proportion of YF NA response in the 3 + 0 PCV/YF separate 9-month group could be due to sub-optimal storage or administration of the YF vaccine. Additionally, the different YF neutralizing assay methods used to determine the YF seroconversion in our study and both studies mentioned above may explain the differences in the proportions of seroprotection reported. It is essential to standardize the neutralization assays to enable the comparison of data across various studies. Furthermore, while we used PCV13 in our trial, both studies mentioned above used PCV10. There is insufficient evidence of the influence of PCV valency on YF vaccine seroresponse and this should be investigated although it is unlikely that there would be a difference from the findings with PCV13.

One unusual finding in our study was the difference in the proportions in the two groups that received YF vaccines separately without PCV, i.e. 81 % in the 3 + 0 PCV/YF separate 9-month group in which YF vaccines were administered together with measles/rubella vaccines at 9 months, and 92 % in the 1 + 1 PCV/YF separate 10-month group in which YF vaccines were administered separately without measles/rubella vaccines in the PP cohort. There is inconsistent evidence regarding the immunogenicity of YF vaccine when co-administered with measles/rubella vaccines. While some studies found no effect on YF seroresponse when co-administered with measles/rubella vaccine, other reports suggest a decrease in the immunogenicity of YF vaccine when co-administered with measles/rubella vaccines. Nascimento Silva et al. found a notable difference in seroresponse rates between administering YF vaccine separately and when co-administered with the measles, mumps, and rubella vaccines (86.5 % vs 69.5 %). [11] Another study also reported similar rates, with 9 to 11-month-old infants who received the YF vaccine co-administered with the measles vaccine showing a seroconversion rate of 72 %. [13] In our study, the proportion of participants with YF NA titre ≥1:8 in the two groups that received the YF vaccines together with measles/rubella vaccines at 9 months were lower, i.e. 81 % in the 3 + 0 PCV/YF separate 9-month group and 85 % in the 1 + 1 PCV/YF co-administration 9-month group in the PP cohort compared to 92 % in the 1 + 1 PCV/YF separate 10-month group which received YF separately without measles/rubella vaccines. This finding in our study could be due to chance because of insufficient sample sizes in the two groups: 43 and 63 samples were available for testing compared to the predetermined sample size of 112 in each group. The baseline characteristics of the 3 + 0 PCV/YF separate 9-month and the 1 + 1 PCV/YF separate 10-month groups were similar (Supplementary table S2), so this finding is unlikely to be due to selection bias. Due to the differences in the age of administration of the YF vaccine in the two groups, a sensitivity analysis adjusted for age showed an insignificant impact on the proportions of YF NA titres ≥1:8 in the groups (Supplementary table S4). Due to limited data, further studies with a design that includes a YF vaccine administered separately comparator may be necessary to gain a better understanding of the potential interference on YF vaccine seroresponse when co-administered with measles/rubella vaccines and other PCVs of varying valencies from different manufacturers.

In our study, there were no reported incidents of reactogenicity following the co-administration of YF vaccine and PCV. However, some local minor reactions might have been missed since the surveillance for reactogenicity was mainly passive.

A strength of our study is that it reflects real-world immunization scenarios in most LMICs. For example, during the trial, there were instances of YF vaccine stock out (on 08 July 2021 and from 07 to 13 September 2021) and a nationwide public health officer's strike on 06 to 10 April 2022 and from 06 June to 04 July 2022. The investigators did not control the vaccine delivery as the existing government EPI structures delivered the vaccines. These incidents resulted in some participants receiving the YF vaccine outside the window period and these participants were excluded from the PP analysis. These occurrences mirror real-world vaccination implementation challenges; thus, our data is more pragmatic and implies effectiveness rather than efficacy.

There are some limitations to the study. Firstly, we failed to collect and test sufficient numbers of specimens according to the predetermined sample size of 112 participants in each arm. This was due to participant withdrawal, refusal of blood collection, and insufficient volume of blood samples. The small sample size in the groups indicates insufficient statistical power to test the non-inferiority hypothesis of the study. To investigate potential selection bias due to missing data, the sensitivity analysis to determine whether the excluded children differ in background characteristics from those who were included in the analyses showed no difference in baseline characteristics across all groups (Supplementary table S3). Additionally, we did not measure the baseline seropositivity of YF before vaccination. Although none of the participants had received YF vaccine or PCV prior to enrolment, information on baseline seroprevalence of YF vaccine would have enabled us to assess the seroconversion rate of YF vaccine in exposed infants. Assuming a baseline YF NA titre ≥8 of around 2.2 % in YF vaccine naive cohort, as reported by a previous study in The Gambia, [10] baseline antibody titre is unlikely to have influenced the overall YF seroconversion rate in our study. Although previous studies have found no difference in seroconversion rates when comparing YF vaccination at 9 months and 12 months, [6,38] our findings may not be generalized to settings that have different YF vaccination schedules to the Gambia's.

## 5 Conclusion

The analyses reported in this paper could not establish the non-inferiority or inferiority of YF vaccine seroresponse when co-administered with PCV13. However, the levels of YF NA attaining seroprotection (NT ≥1:8) were high and robust in all groups. A higher percentage of seropositivity would be optimum and might be achieved through improving overall EPI coverage or by giving a second dose. PCV13 co-administered with YF vaccine at 9 months does not affect seroresponse to YF vaccine, and co-administration can be recommended if this fits into the schedule of a national EPI programme.

## Funding

This work was supported by the 10.13039/100000865Bill & Melinda Gates Foundation [grant number OPP1138798]. The funders had no role in the study design, data analysis, data collection, data interpretation or writing of the report. The PI and corresponding author had full access to all the data in the study and had final responsibility for the decision to submit the paper for publication.

## CRediT authorship contribution statement

**Isaac Osei:** Writing – review & editing, Writing – original draft, Methodology, Investigation, Formal analysis, Conceptualization. **Jonas Schmidt-Chanasit:** Writing – review & editing, Supervision, Investigation, Conceptualization. **Paul V. Licciardi:** Writing – review & editing, Investigation. **Ousman Secka:** Writing – review & editing, Investigation. **Umberto D'Alessandro:** Writing – review & editing, Investigation. **Rasheed Salaudeen:** Writing – review & editing, Investigation. **Golam Sarwar:** Writing – review & editing, Investigation, Data curation. **Ed Clarke:** Writing – review & editing, Investigation. **Nuredin I. Mohammed:** Writing – review & editing, Supervision, Formal analysis. **Cattram Nguyen:** Writing – review & editing, Supervision, Formal analysis. **Brian Greenwood:** Writing – review & editing, Supervision, Investigation. **Stephanie Jansen:** Writing – review & editing, Methodology, Investigation, Data curation. **Grant A. Mackenzie:** Writing – review & editing, Validation, Supervision, Resources, Methodology, Investigation, Funding acquisition, Data curation, Conceptualization.

## Declaration of competing interest

The authors declare the following financial interests/personal relationships which may be considered as potential competing interests:

Grant Mackenzie reports financial support was provided by Bill & Melinda Gates Foundation. If there are other authors, they declare that they have no known competing financial interests or personal relationships that could have appeared to influence the work reported in this paper.

## Data Availability

Data will be made available on request.

## Appendix A Supplementary data

**Supplementary Table S1**. Trial groups and timeline of vaccination, specimen collection, and measurement of endpoints Image 1

Supplementary material 2 Image 2

Supplementary material 3 Image 3

Supplementary material 4 Image 4
