# Supplementary material for: Immunogenicity of yellow fever vaccine co-administered with 13-valent pneumococcal conjugate vaccine in rural Gambia: A cluster-randomised trial
Source: Vaccine. 2025 Feb 15;47:None. doi: 10.1016/j.vaccine.2025.126712 (PMC11797555; doi:10.1016/j.vaccine.2025.126712)
Supplement: Supplementary file 1 — Supplementary Table S1. Trial groups and timeline of vaccination, specimen collection, and measurement of endpoints [file mmc1.docx]

**Supplementary Table S1.**

**Trial groups and timeline of vaccination, specimen collection, and measurement of endpoints**

|  | **Group** | | |
| --- | --- | --- | --- |
| **Age** | **3+0**  **PCV/YF separate 9-month** | **1+1**  **PCV/YF co-administration 9-month** | **1+1**  **PCV/YF separate 10-month** |
| **6 wk** | ***PCV13***  ***NPS*** | ***PCV13***  ***NPS*** | ***PCV13***  ***NPS*** |
| **10 wk** | ***PCV13*** | ***Blood***  IgG  OPA |  |
| **14 wk** | ***PCV13*** |  |  |
| **18 wk** | ***Blood***  IgG  OPA |  |  |
| **9 mo** | ***YF***  ***Blood***  IgG  ***NPS*** | ***YF & PCV13***  ***Blood***  IgG  ***NPS*** | ***PCV13***  ***Blood***  IgG  ***NPS*** |
| **10 mo** | ***Blood***  YFNA  IgG  OPA  ***NPS*** | ***Blood***  YFNA  IgG  OPA  ***NPS*** | ***YF***  ***Blood***  IgG  ***NPS*** |
| **11 mo** | ***NPS*** | ***NPS*** | ***Blood***  YFNA  ***NPS*** |

PCV13; 13-valent pneumococcal conjugate vaccine

YF; yellow fever vaccine

IgG; serotype-specific anti-pneumococcal IgG

YFNA; yellow fever neutralizing antibody

OPA; opsonophagocytic assay
