# Supplementary material for: Immunogenicity of yellow fever vaccine co-administered with 13-valent pneumococcal conjugate vaccine in rural Gambia: A cluster-randomised trial
Source: Vaccine. 2025 Feb 15;47:None. doi: 10.1016/j.vaccine.2025.126712 (PMC11797555; doi:10.1016/j.vaccine.2025.126712)
Supplement: Supplementary file 2 — Supplementary material 2 [file mmc2.docx]

**Supplementary Table S2.**

**Baseline Characteristics comparing all participants who had samples available and those with missing samples, by Group**

| **Characteristic** | **Group** | | | | | | | |
| --- | --- | --- | --- | --- | --- | --- | --- | --- |
|  | **3+0 PCV/YF separate**  **9-month** | | **1+1 PCV/YF co-administration 9-month** | | **1+1 PCV/YF separate**  **10-month** | | **1+1 PCV/YF separate and 3+0 PCV** | |
|  | Available samples | Missing samples | Available samples | Missing samples | Available samples | Missing samples | Available samples | Missing samples |
| No. enrolled | 48 | 64 | 66 | 52 | 98 | 18 | 146 | 82 |
| Age at enrolment (days), n | 48 | 64 | 66 | 52 | 98 | 18 | 146 | 82 |
| median (IQR), n | 21 (11 - 33) | 27 (18 - 40) | 22 (15 - 34) | 31 (17 – 40) | 27 (17 - 43) | 28 (21 – 38) | 24 (16 -39) | 28 (18 – 40) |
| Sex, n | 48 | 64 | 66 | 52 | 98 | 18 | 146 | 82 |
| female, n (%) | 23 (48%) | 31 (48%) | 30 (45%) | 24 (46%) | 53 (54%) | 7 (39%) | 76 (52%) | 38 (46%) |
| Gestational age at birth, n | 48 | 64 | 66 | 52 | 98 | 18 | 146 | 82 |
| median (IQR), n | 38 (37 - 38) | 38 (37 – 38) | 38 (37 - 38) | 38 (37 – 38) | 38 (37 - 38) | 38 (37 – 38) | 38 (37 - 38) | 38 (37 – 38) |
| ^#^Birth weight, n | 37 | 56 | 52 | 46 | 86 | 17 | 123 | 73 |
| median (IQR), n | 3.0 (2.8 - 3.3) | 3.1 (2.7 – 3.4) | 3.1 (3.0 - 3.5) | 3.0 (3.0 – 3.5) | 3.0 (2.7 - 3.3) | 3.2 (3.0 – 3.5) | 3.0 (2.8 - 3.3) | 3.1 (2.8 – 3.4) |
| Breastfed at enrolment, n | 48 | 64 | 66 | 52 | 98 | 18 | 146 | 82 |
| yes, n (%) | 48 (100%) | 64 (100%) | 66 (100%) | 52 (100%) | 98 (100%) | 18 (100%) | 146 (100%) | 82 (100%) |
| Age at first PCV dose (days), n | 48 | 64 | 66 | 52 | 98 | 18 | 146 | 82 |
| median (IQR), n | 60 (50 - 67) | 58 (51 – 68) | 55 (50 - 63) | 61 (48 – 69) | 56 (48 - 65) | 63 (52 – 85) | 56 (49 - 65) | 59 (51 – 70) |
| Age at second PCV dose (days), n | 48 | 64 | 66 | 51 | 98 | 18 | 146 | 82 |
| median (IQR), n | 90 (83 - 101) | 98 (84 – 111) | 295 (283 - 311) | 288 (281 – 305) | 292 (283 - 301) | 295 (280 – 304) | NA | NA |
| Age at third PCV dose (days), n | 48 | 64 | NA | NA | NA | NA | NA | NA |
| median (IQR), n | 127 (118 - 144) | 133 (125 – 154) | NA | NA | NA | NA | NA | NA |
| Age at YF vaccine dose (days), n | 48 | 55 | 66 | 50 | 98 | 18 | 0 | 9 |
| median (IQR), n | 292 (284 - 310) | 295 (281 – 304) | 296 (284 - 311) | 288 (282 – 306) | 327 (310 - 356) | 332 (301 – 352) | 319 (290 - 348) | 298 (285 – 330) |
| Antibiotics since birth, n | 48 | 63 | 66 | 52 | 98 | 18 | 146 | 81 |
| yes, n (%) | 4 (8%) | 5 (8%) | 1 (1%) | 3 (6%) | 6 (6%) | 5 (28%) | 10 (7%) | 10 (12%) |
| Smoker in house, n | 47 | 63 | 66 | 52 | 98 | 18 | 145 | 81 |
| yes, n (%) | 3 (6%) | 6 (9%) | 11 (17%) | 7 (13%) | 14 (14%) | 0 (0%) | 17 (12%) | 6 (7%) |
| Household cooking fuel, n | 44 | 63 | 63 | 52 | 89 | 18 | 133 | 82 |
| wood, n (%) | 42 (95%) | 62 (98%) | 60 (95%) | 51 (98%) | 87 (98%) | 18 (100%) | 129 (97%) | 81 (99%) |
| charcoal, n (%) | 2 (5%) | 1 (2%) | 3 (5%) | 1 (2%) | 2 (2%) | 0 (0%) | 4 (3%) | 1 (1%) |
| Infants inside the cooking area sometimes, n | 44 | 62 | 63 | 52 | 89 | 18 | 133 | 81 |
| yes, n (%) | 21 (48%) | 30 (48%) | 35 (55%) | 25 (48%) | 47 (53%) | 8 (44%) | 68 (51%) | 38 (47%) |

# These births occurred at home, so newborns were not weighed at birth

NA; Not Applicable

3+0 PCV/YF separate 9-month– Three early doses of PCV13 scheduled at 6,10, and 14 weeks and Yellow Fever/Measles/Rubella vaccines at 9 months of age

1+1 PCV/YF co-administration 9-month– PCV13 was given at 6 weeks and Yellow Fever vaccine was given together with PCV13 and Measles/Rubella vaccines at 9 months of age

1+1 PCV/YF separate 10-month– PCV13 was given at 6 weeks and 9 months and Yellow Fever vaccine was given separately at 10 months of age

1+1 PCV/YF separate and 3+0 PCV– Yellow fever vaccines were given separately at 10 months and 9 months of age respectively without PCV13.
