# Supplementary material for: Immunogenicity of yellow fever vaccine co-administered with 13-valent pneumococcal conjugate vaccine in rural Gambia: A cluster-randomised trial
Source: Vaccine. 2025 Feb 15;47:None. doi: 10.1016/j.vaccine.2025.126712 (PMC11797555; doi:10.1016/j.vaccine.2025.126712)
Supplement: Supplementary file 3 — Supplementary material 3 [file mmc3.docx]

S1. Baseline characteristics of all participants assigned to Immunogenicity

| Characteristics | Group | | | |
| --- | --- | --- | --- | --- |
|  | 3+0 PCV/YF separate 9-month | 1+1 PCV/YF co-administration 9-month | 1+1 PCV/YF separate 10-month | 1+1 PCV/YF co-administration 9-month and 1+1 YF/PCV separate |
| No. enrolled | 112 | 118 | 117 | 235 |
| Age at enrolment (days), n | 112 | 118 | 117 | 235 |
| median (IQR), n | 25 (15 - 37) | 26 (16 -36) | 27 (18 - 43) | 27 (17 - 39) |
| Sex, n | 112 | 118 | 117 | 235 |
| female, n (%) | 54 (48%) | 54 (46%) | 59 (50%) | 113 (48%) |
| Gestational age at birth, n | 112 | 118 | 117 | 235 |
| median (IQR), n | 38 (37 - 38) | 38 (37 - 38) | 38 (37 - 38) | 38 (37 - 38) |
| ^#^Birth weight, n | 93 | 99 | 100 | 199 |
| median (IQR), n | 3.0 (2.8 - 3.3) | 3.1 (3.0 - 3.5) | 3.0 (2.8 - 3.4) | 3.0 (2.9 - 3.4) |
| Breastfed at enrolment, n | 112 | 118 | 117 | 235 |
| yes, n (%) | 112 (100%) | 118 (100%) | 117 (100%) | 235 (100%) |
| Age at first PCV dose (days), n | 112 | 118 | 117 | 235 |
| median (IQR), n | 59 (50 – 67) | 56 (49 - 65) | 57 (49 - 65) | 57 (49 - 65) |
| Age at second PCV dose (days), n | 112 | 117 | 117 | 234 |
| median (IQR), n | 95 (84 - 108) | 291 (282 - 306) | 293 (282 - 301) | 292 (106 - 298) |
| Age at third PCV dose (days), n | 111 | 118 | 117 | 235 |
| median (IQR), n | 131 (121 - 152) | 126 (124 - 136) | 128 (128 - 128) | 127 (125 - 132) |
| Age at Yellow fever vaccine dose (days), n | 102 | 116 | 116 | 232 |
| median (IQR), n | 293 (283 - 307) | 292 (283 - 308) | 328 (310 - 355) | 309 (286 - 336) |
| Antibiotics since birth, n | 111 | 118 | 117 | 235 |
| yes, n (%) | 9 (8%) | 4 (3%) | 11 (9%) | 15 (6%) |
| Smoker in house, n | 110 | 118 | 117 | 235 |
| yes, n (%) | 9 (8%) | 18 (15%) | 14 (12%) | 32 (14%) |
| Household cooking fuel, n | 108 | 115 | 108 | 223 |
| wood, n (%) | 106 (98%) | 112 (97%) | 106 (98%) | 218 (98%) |
| charcoal, n (%) | 2 (2%) | 3 (3%) | 2 (2%) | 5 (2%) |
| Infant inside cooking area sometimes, n | 108 | 115 | 108 | 223 |
| yes, n (%) | 52 (48%) | 61 (53%) | 54 (50%) | 115 (51%) |
| Age post YF vaccine blood collection (days), n | 48 | 66 | 98 | 164 |
| median (IQR), n | 318 (313 - 335) | 328 (315 - 364) | 360 (349 - 380) | 353 (328 - 382) |

# These births occurred at home, so newborns were not weighed at birth

NA; Not Applicable

3+0 PCV/YF separate 9-month– Three early doses of PCV13 scheduled at 6,10, and 14 weeks and Yellow Fever/Measles/Rubella vaccines at 9 months of age

1+1 PCV/YF co-administration 9-month– PCV13 was given at 6 weeks and Yellow Fever vaccine was given together with PCV13 and Measles/Rubella vaccines at 9 months of age

1+1 PCV/YF separate 10-month– PCV13 was given at 6 weeks and 9 months and Yellow Fever vaccine was given separately at 10 months of age

1+1 PCV/YF co-administration 9-month and 1+1 YF/PCV separate – All participants assigned to the 1+1 schedule; PCV give at 6weeks and 9 months
