# Supplementary material for: Immunogenicity of yellow fever vaccine co-administered with 13-valent pneumococcal conjugate vaccine in rural Gambia: A cluster-randomised trial
Source: Vaccine. 2025 Feb 15;47:None. doi: 10.1016/j.vaccine.2025.126712 (PMC11797555; doi:10.1016/j.vaccine.2025.126712)
Supplement: Supplementary file 4 — Supplementary material 4 [file mmc4.docx]

**Supplementary table S3**

**Yellow Fever neutralizing antibody titres ≥1:8 1 month post-vaccination adjusted for age at YF vaccination**

| Study group | n/N | Percentage (95% CI) participants Yellow Fever neutralizing antibody titre ≥1:8 | Difference in percentages (95% CI) compared to YF/PCV Co-administration |
| --- | --- | --- | --- |
| Per Protocol (PP) | | | |
| 1+1 PCV/YF co-administration 9mo | 51/60 | 84% (76% to 93%) | 1 (reference) |
| 3+0 PCV/YF separate 9mo | 35/43 | 82% (73% to 91%) | 2% (-10% to 15%) |
| 1+1 PCV/YF separate 10mo | 58/63 | 92% (84% to 99%) | -6% (-18% to 6%) |
| 1+1 YF/PCV separate and 3+0 | 93/106 | 88% (80% to 93%) | -4% (-13% to 8%) |
| Intention to treat (ITT) | | | |
| 1+1 PCV/YF co-administration 9mo | 56/66 | 84% (74% to 92%) | 1 (reference) |
| 3+0 PCV/YF separate 9mo | 38/48 | 79% (65% to 90%) | 5% (-8% to 18%) |
| 1+1 PCV/YF separate 10mo | 88/98 | 90% (82% to 95%) | -6% (-14% to 2%) |
| 1+1 PCV/YF separate and 3+0 PCV | 126/146 | 87% (80% to 91%) | -3% (-12% to 6%) |

ITT – Intention to treat

PP – Per protocol

3+0 PCV/YF separate 9-month– Three early doses of PCV13 scheduled at 6,10, and 14 weeks and Yellow Fever/Measles/Rubella vaccines at 9 months of age

1+1 PCV/YF co-administration 9-month– PCV13 was given at 6 weeks and Yellow Fever vaccine was given together with PCV13 and Measles/Rubella vaccines at 9 months of age

1+1 PCV/YF separate 10-month– PCV13 was given at 6 weeks and 9 months and Yellow Fever vaccine was given separately at 10 months of age

1+1 PCV/YF separate and 3+0 PCV– Yellow fever vaccines were given separately at 10 months and 9 months of age respectively without PCV13.
